# Aryl Polyphosphonates: Useful Halogen-Free Flame Retardants for Polymers

**DOI:** 10.3390/ma3104746

**Published:** 2010-10-11

**Authors:** Li Chen, Yu-Zhong Wang

**Affiliations:** Center for Degradation and Flame-Retardant Polymeric Materials, College of Chemistry, State Key Laboratory of Polymer Materials Engineering, National Engineering Laboratory of Eco-Friendly Polymeric Materials (Sichuan), Sichuan University, Chengdu 610064, China; E-Mail: l.chen.scu@gmail.com (L.C.)

**Keywords:** aryl polyphosphonates, flame retardant, thermal stability, mechanism

## Abstract

Aryl polyphosphonates (ArPPN) have been demonstrated to function in wide applications as flame retardants for different polymer materials, including thermosets, polycarbonate, polyesters and polyamides, particularly due to their satisfactory thermal stability compared to aliphatic flame retardants, and to their desirable flow behavior observed during the processing of polymeric materials. This paper provides a brief overview of the main developments in ArPPN and their derivatives for flame-retarding polymeric materials, primarily based on the authors’ research work and the literature published over the last two decades. The synthetic chemistry of these compounds is discussed along with their thermal stabilities and flame-retardant properties. The possible mechanisms of ArPPN and their derivatives containing hetero elements, which exhibit a synergistic effect with phosphorus, are also discussed.

## 1. Introduction

For many years, versatile phosphorus-containing compounds with several oxidation states have been widely known as flame retardants for polymer materials [1]. Among them, elemental red phosphorus, phosphines, phosphine oxides, phosphites, phosphonates and phosphates all performed broad applications of flame retardants [2,3,4,5]. These phosphorus-containing flame retardants can be utilized as additives or incorporated into the polymer chain during its polymerization, and are known to be active in the condensed and/or gaseous phase, depending on the chemical nature and thermal stability of the additives as well as the host polymer matrices [6].

**Scheme 1:**
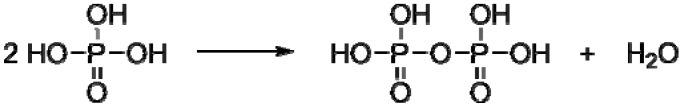
Pyrophosphate structure formed from phosphoric acid condensation.

**Scheme 2:**
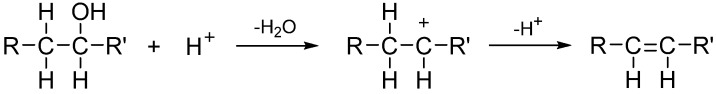
Formation of double carbon-carbon bonds after the dehydration of alcohol end groups.

In the condensed phase, the phosphorus-containing flame retardants are selectively active with the host polymers containing oxygen (*i.e*., polyesters, polyamides, cellulose, *etc*.) during heating or combustion [7]. With most of the phosphorus-containing flame retardants, thermal decomposition leads to the production of phosphoric acid, which condenses readily to produce pyrophosphate structures and release water vapor (see **Scheme 1**). The water released can dilute the oxidizing and combustible gas phases. In addition, phosphoric acid and pyrophosphoric acid can catalyze the dehydration reaction of the alcohol groups, leading to the formation of carbocations and carbon-carbon double bonds (see **Scheme 2**), and consequently to the aromatization at high temperature. At high temperature, ortho- and pyrophosphoric acids are turned into metaphosphoric acid [(O)P(O)(OH)] and their corresponding polymers [(PO_3_H)_n_]. The phosphate anions (*i.e*., pyro- and polyphosphates) then participate with the carbonized residues in char formation. This carbonized layer (char residues) can isolate and protect the polymer from the flames, limit the volatilization of fuel, prevent the formation of new free-radicals, confine the oxygen diffusion to reduce combustion, and insulate the polymer underneath from the heat.

However, due to their inherent drawbacks, including the potential safety hazard of red phosphorus during compounding with polymer matrices, reduced hydrolytic stability and plasticizing effect, particularly caused by the small-molecular and oligomeric phosphorus-containing organics, these compounds are undesirable in many application fields. Also, relative high volatilization and migration of the additives during compounding and/or occupation could further limit the wide application of the small-molecular and oligomeric phosphorus-containing organics. Consequently, polymer-type phosphates and phosphonates have received increasing interests from both academic and industrial fields.

Compared with aliphatic polyphosphonates (AlPPN) and aromatic polyphosphates (ArPPA), aryl polyphosphonates (ArPPN) usually exhibit better thermal stability than AlPPN during processing and molding, and higher hydrolytic stability than ArPPA due to the partly hydrolyzable P-O-C bond substituted by the hydrophobic P-C bond [8]. Therefore, applications of ArPPN in flame retardation are more prevalent than the others, particularly for aromatic polycondensates with high processing temperature.

## 2. Main-Chain Phosphorus-Containing Aryl Polyphosphonates

**Scheme 3:**
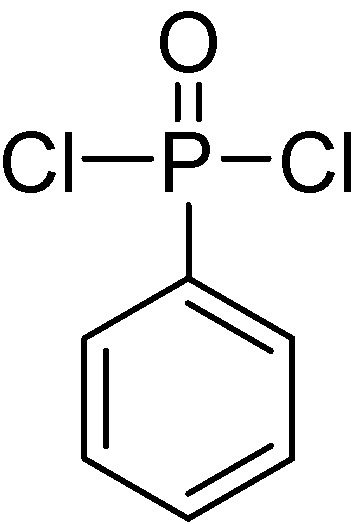
Chemical structure of phenylphosphonic dichloride (PPC).

**Scheme 4:**
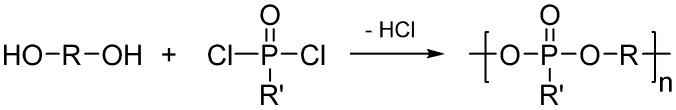
Synthesis route of main chain aromatic polyphosphonates (MC-ArPPN)

Phenylphosphonic dichloride (PPC, **Scheme 3**) is a typical intermediate to prepare main-chain phosphorus-containing materials, due to its two highly reactive phosphorylchlorides, which can easily react with the compounds containing hydroxy or amino groups. Based on this, Stackman [9] has synthesized a series of main chain aromatic polyphosphonates (MC-ArPPN, see **Scheme 4**) derived from PPC and diphenols, and examined the effects of structural variations and molecular weights of target MC-ArPPNs upon their flame-retardant activity in aromatic polyesters, including poly(ethylene terephthalate) (PET) and poly(butylene terephthalate) (PBT). A series of diphenolic monomers, including resorcinol, hydroquinone and bisphenol A, were employed into the main chain of the target MC-ArPPN. Detailed results are listed in Table 1 and Table 2. From the data listed in Table 1, it can be concluded that all of these ArPPNs showed flame-retardant activity to some degree; however, on a weight basis the resorcinol (sample 1) and hydroquinone (sample 2) based ArPPNs exhibited superior performance due to the higher phosphorus content of the polyphosphonate (*i.e*., 13 wt % for samples 1 and 2 *vs.* 8.8 wt % for sample 3). The author also investigated the flame-retardant activity of poly(1,3-phenylene phenylphosphonate) (PPP) with different molecular weights on both PET and PBT (Table 2); however, the results indicate that there is no discernible difference in flame-retardant performance, as measured by LOI value, between the lower and the higher molecular weight PPP. Nevertheless, there may be some advantages in physical property retention due to the use of a high molecular weight additive. Furthermore, these kinds of MC-ArPPN with extremely high molecular weight were considered to be functional materials, which could be drawn into long fine fibers of remarkable strength, or be drawn out in thin translucent sheets similar to the cellophane in appearance and flexibility [10].

**Scheme 5:**
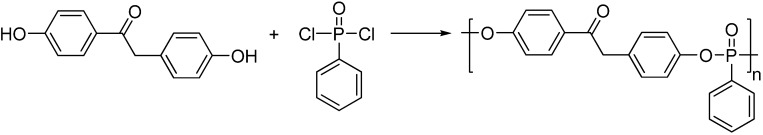
Synthesis route of BHDB-polyphosphonate (BHDB-PPN).

Ranganathan *et al*. [11] prepared a novel MC-ArPPN, BHDB-PPN (see **Scheme 5**) based on PPC and 4,4’-bishydroxydeoxybenzoin (BHDB) as the diphenolic monomer through interfacial polycondensation. Pyrolysis combustion flow calorimetry (PCFC) [12] was employed to evaluate the flame-retardant properties of the target polyphosphonates, and the results are summarized in Table 3.

**Table 1:** Effect of structural variations on flame-retardant activity of MC-ArPPN in PET and PBT [9].

No.	R	R'	MC-ArPPN content (wt %)	Properties of the PET blends		Properties of the PBT blends	
				P content (wt %)	LOI (vol%)	P content (wt %)	LOI (vol%)
0			0	0	17.0	0	16.5
1	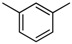	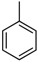	5	0.54	18.6	0.60	18.7
			10	1.30	21.0	1.27	19.4
2	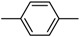	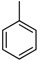	5	0.48	18.9	0.64	18.1
			10	1.53	22.0	1.17	19.4
3	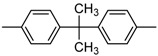	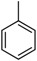	10	1.09	18.8	0.83	18.3

**Table 2:** Effect of molecular weight of poly(1,3-phenylene phenylphosphonate) (PPP) on flame retardance [9].

Matrix	PPP content (wt %)	Intrinsic viscosity of PPP (dL/g)	Phosphorus content (wt %)	LOI (vol %)
PET	5	0.46	0.52	18.6
	10	0.46	1.16	19.9
	5	0.17	0.54	18.6
	10	0.17	1.33	20.0
PBT	5	0.46	0.58	18.7
	10	0.46	1.19	20.4
	5	0.17	0.66	18.7
	10	0.17	1.30	20.7

**Scheme 6:**
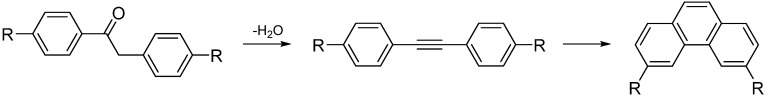
Potential route to aromatization of BHDB segments.

It could be concluded that BHDB-PPN has exceptionally low heat release capacity (HRC) value compared to many existing inherently flame-retardant polymers such as poly(phenyl sulfone) (PPS) and Vetra C LCP. The authors suggested the potential utility to promote char forming in burning polymer materials, arising from the elimination of H_2_O on BHDB segment at the presence of phosphorus-containing acids (formed during pyrolysis of phenylphosphonic segment) to form diphenylacetylene, which could further undergo rearrangement to aromatization (see **Scheme 6**) [13]. Increased charring residues would enhance the flame retardancy of the target polyphosphonate. The authors did not apply BHDB-PPN into other polymers as the flame-retardant additive; however, due to its high thermal stability (5 wt % mass loss observed at 345 °C) and char yield, BHDB-PPN could be employed as an additive to flame-retard polymers and exhibit flame-retardant action in condensed phase.

**Table 3:** Measured heat release capacities of BHDB-PPN and other reference materials.

Sample	Heat release capacity (J/(g.K))	Char yield at 800 °C (wt %)
BHDB-PPN	81 ± 11	52.0
polyethylene	1676	0
polystyrene	927	0
polycarbonate (BPA)	359	21.7
poly(ethylene terephthalate)	332	5.1
poly(vinyl chloride)	138	15.3
poly(2,6-dimethylene 1,4-phenyleneoxide)	409	25.5
poly(phenyl sulfone)	153	38.4
Vectra C LCP ^a^	164	40.6

^a^ Vectra C LCP is a commercial liquid crystalline copolyester of hydroxybenzoic and hydroxynapthoic acids from Hoechst Celanese.

**Scheme 7:**
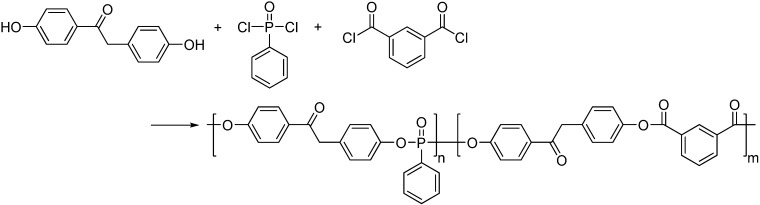
Synthesis route of BHDB-poly(arylate-*co*-phosphonate) (PAL-*co*-PPN).

Ranganathan *et al*. [14] have introduced isophthaloyl chloride (iPC) into BHDB-PPN to form the BHDB-poly(arylate-*co*-phosphonate) copolymers (PAL-*co*-PPN, see **Scheme 7**) through solution polycondensation. Results of flammability and thermal stability of the prepared copolymers and homopolymers, evaluated by PCFC and thermogravimetric analysis (TGA), respectively, are listed in Table 4. Interestingly, all of the PAL-*co*-PPN copolymers exhibited even lower HRC values than the two homopolymers. For example, the copolymer with a composition of approximately 1:1 arylate:phosphonate had the lowest HRC value of 36 J/(g.K); however, the arylate homopolymer exhibited a HRC value of 65 J/(g.K) and the phosphonate homopolymer exhibited a highest HRC value of 81 J/(g.K). Char yields of the copolymers at 800 °C were also higher than those of both homopolymers. Seemingly, the flammability and thermal stability results were not in accord with the phosphorus contents of the PAL-*co*-PPN copolymers.

The authors suggested that the higher oxygen content of PAL-*co*-PPN copolymers, due to the isophthalate and BHDB moieties, should be responsible for the highest char yields and lowest HRC values of all the polymers. Pyrolysis gas-chromatography/mass spectrometry (Py-GC/MS) results of these polymers indicated that no phosphorous-containing compounds were present in the volatile decomposition products of BHDB-ArPPN and PAL-*co*-PPN copolymers [15]. Hence, a condensed phase mechanism that could depress the HRC value in BHDB-ArPPN and PAL-*co*-PPN copolymers appeared to be operational here.

**Table 4:** Flammability and thermal stability characterization of BHDB-poly(arylate-*co*-phosphonate) copolymers [15].

iPC : PPC (molar ratio)	Heat release capacity (J/(g.K))	Thermal stability parameters ^a^	
		5 wt % loss temperature (°C)	Char yield at 800 °C (wt %)
100:0	65 ± 5	340	45
77:23	48 ± 4	346	50
57:43	41 ± 3	383	56
46:54	36 ± 2	367	54
39:61	40 ± 3	390	57
23:77	59 ± 5	394	55
0:100	81 ± 11	397	52

^a^ thermal stability parameters were obtained from TGA.

**Scheme 8:**
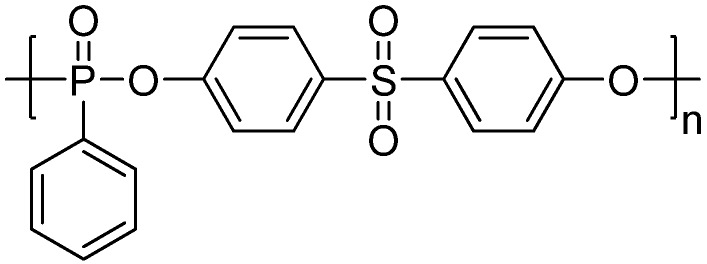
Chemical structure of poly(sulfonyldiphenylene phenylphosphonate) (PSPPP).

Sulfur is another flame retardant element which may act as the acid source in the intumescent flame retardant system [16]. A sulfur-containing MC-ArPPN, named poly(sulfonyldiphenylene phenylphosphonate) (PSPPP, **Scheme 8**) was synthesized successfully through melt [17] or solution polycondensation [18,19,20] between phenylphosphonic dichloride and bisphenol S (4,4’-sulfonyldiphenol).

PSPPP has been used to prepare the flame-retardant terylene by adding it to PET before spinning for many years [18,20,21]. Granzow pointed out that the choice of phosphorus-containing flame retardants in PET should be restricted to those which are thermally stable during the processing temperature of PET around 300 °C; but to be effective, the additive has to decompose rapidly around 400 °C, the surface temperature of burning polyester [22]. Aside from these thermal stability constraints, good compatibility with the polyester melt and the absence of any detrimental effects on the spinnability are mandatory. PSPPP have been found to be particularly suitable for the spinning of PET and to meet those various requirements [18,20].

Since 1987, Wang and co-workers have begun to systematically investigate PSPPP and its applications [21,23,24], including new synthetic methods [17], toxicity [25], solubility parameters [24], miscibility with PET [24], preparation of the flame-retardant masterbatch [26], and its effects on the rheological properties [27], the crystallizability [28], the spinability [29], and the fiber dyeability [30] of PET. The thermo-oxidative degradation behaviors [31] and flame-retardant mechanism of PET/PSPPP systems were also investigated. Results suggested that PSPPP is a highly efficient additive-type flame retardant for PET: 5 wt % of PSPPP can increase the LOI of PET from 21 to 30, and achieve a UL-94 V-0 rating. TGA results indicated that the activation energy for the decomposition of PSPPP at the early stage of thermal degradation is higher than that of PET, and the decomposition temperature ranges of PSPPP and PET overlapped with each other, which could meet the thermal stability requirement as above-mentioned [22]. Furthermore, the flame-retardant PET fibers and plastics obtained using PSPPP as flame retardants have much better comprehensive performance than other low molecular flame retardants, including lower toxicity, better miscibility, crystallizability, spinability and fiber dyeability, higher thermal stability and flame retardancy. PSPPP is also a good flame retardant for polyamides and other polyesters [32].

**Scheme 9:**
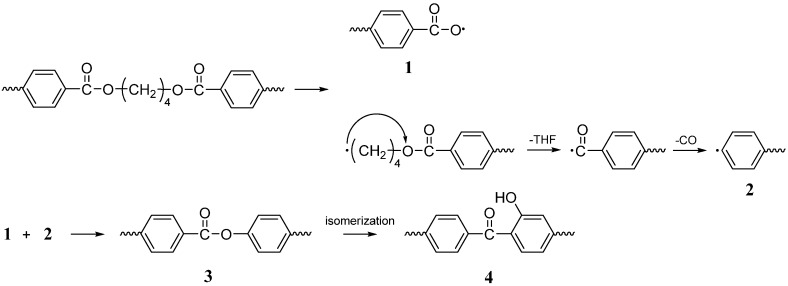
Formation of polyarylates (3) and phenolic structures (4) on the pyrolysis of PBT modified by PSPPP.

**Scheme 10:**
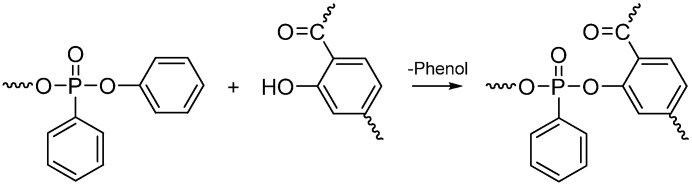
Formation of P-O-C crosslink structures on the pyrolysis of PBT modified by PSPPP.

Balabanovich *et al*. [33] investigated the flame retardancy and charring effect of PSPPP in PBT alone or in combination with polyphenylene oxide (PPO) or 2-methyl-1,2-oxaphospholan-5-one-2-oxide (OP). The UL-94 test V-0 rating could be achieved by addition of 10 wt % PSPPP, 10 wt % PPO and 10 wt % OP. The fire retardant effect was attributed to promoting char yield by involving the polymer in charring. Py-GC/MS results suggested that PSPPP was shown to induce the formation of thermally stable polyarylates and phenolic functionalities in PBT (see **Scheme 9**), and PSPPP was likely to react with these functionalities and crosslink structures by its reactive P-O-Ph and P-Ph bonds (see **Scheme 10**). In connection with this, PPO was employed into the flame-retardant system as an effective co-additive for PSPPP due to producing phenolic structures upon pyrolysis [34]. The phenolic moieties generated may react with the P-O-C of PSPPP and therefore this additive shows a condensed phase activity in PBT modified with PSPPP and PPO. Results suggested the synergistic effect of the condensed-phase-active phosphorus flame retardant (PSPPP) with a vapor-phase-active flame retardant (OP). By the combination of these two modes of flame-retardant action, it is possible to achieve the desirable UL-94 V-0 rating for PBT, which is not succeeded by adding PSPPP or OP alone.

Furthermore, Wang and co-workers [35] investigated the synergistic effect of PSPPP with potassium diphenyl sulfonate (SSK) in polycarbonate (PC). Potassium sulfonates are often used at a very low loading to flame-retard PC [36,37,38], and allow the desired transparency; however, they can cause some hazing of PC [39]. The flame-retardant systems containing PSPPP and SSK exhibited effective synergism: 0.5 wt % SSK plus 4.0 wt % PSPPP endowed PC with a highest LOI value of 36.8 and a UL-94 V-0 rating. The detailed results from LOI and UL-94 tests are listed in Table 5.

**Table 5:** Flame retardancy of PC/PSPPP/SSK mixtures with different contents of the flame retardants [35].

PC (wt %)	Flame retardants (wt %)		Flame retardancy		Charyield at 700 °C (wt %)
	PSPPP	SSK	LOI	UL-94 rating	
100	0	0	26.3	V-2	
95	5	0	31.9	V-2	
95	4.5	0.5	36.8	V-0	18.2
95	4	1	35.2	V-0	20.3
95	2	3	32.9	V-0	16.5
95	0	5	33.0	V-0	

**Figure 1:**
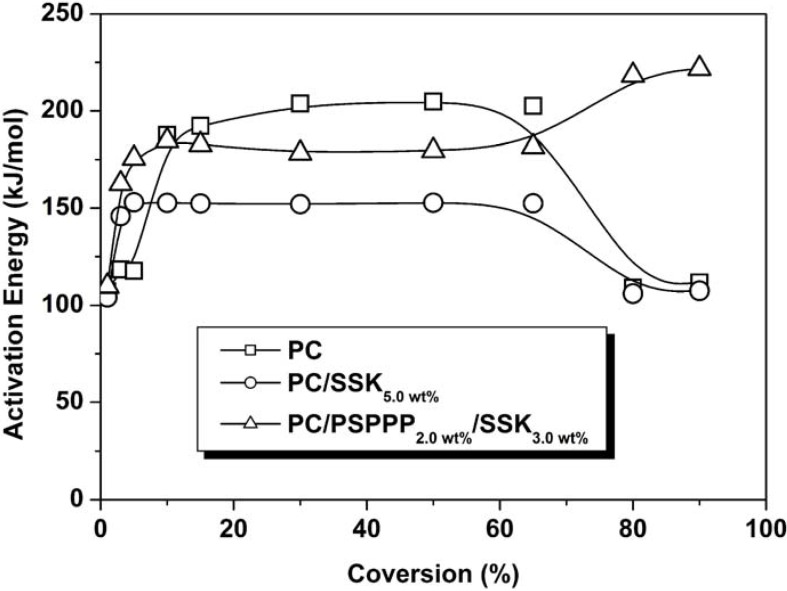
Activation energy (*E_α_*) curves of neat PC and flame retardant PCs calculated according to Flynn method [35].

The resulting data, as well as the analysis of the activation energies characterized by TGA under dynamic conditions, demonstrated that the additives accelerate the thermal degradation of PC, especially in the early stage, by accelerating the evolution of incombustible gas (*i.e*., carbon dioxide); and different additives caused different process in the final stage. Because of the different components of the flame retardants, the variation trend of activation energies for flame-retardant PCs containing 5 wt % SSK alone and combination of SSK (3 wt %) with PSPPP (2 wt %) are significantly different in the final stage (80~90 wt % weight loss), as shown in Figure 1. For SSK + PSPPP system, the increased activation energy in the final stage suggested a charring process, which showed quite an agreement with the increase of LOI value after PSPPP was added.

**Scheme 11:**
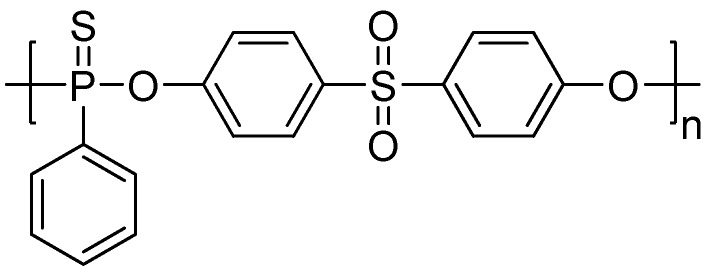
Chemical structure of poly(sulfonyldiphenylene thiophenylphosphonate) (PSTPP).

Further, a novel aryl polyphosphonate derived from PSPPP, poly(sulfonyldiphenylene thiophenylphosphonate) (PSTPP, **Scheme 11**) was also synthesized and used for flame-retarding PET by Wang and co-workers [40,41,42]. The authors suggested that, when the oxygen in P=O was substituted by sulfur, the flame retardant efficiency of the aryl polyphosphonate on PET seemed to become a little worse; however, the anti-dripping behavior of PET was improved. For example, when the phosphorus content reached 2.5 wt %, PET samples achieved UL-94 V-0 rating and a LOI value of 29.4, and no melt dripping was observed. Seemingly, the results from LOI value and UL-94 V rating suggested that the flame-retardant efficiency of PSTPP in PET was quite lower than PSPPP; however, melt-dripping behavior of the samples containing PSTPP was greatly suppressed, suggested a flame-retardant mechanism different from that of PSPPP.

## 3. Main-Chain/Side-Chain Combined Phosphorus-Containing Aryl Polyphosphonates

**Scheme 12:**
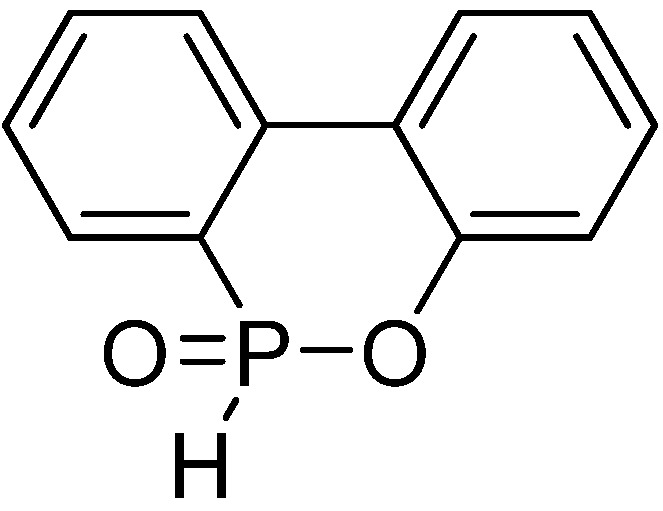
Chemical structure of 9,10-dihydro-9-oxa-phosphaphenthrene-10-oxide (DOPO).

DOPO (9,10-dihydro-9-oxa-phosphaphenthrene-10-oxide, **Scheme 12**) and its derivates were firstly synthesized by Saito [43] in 1972 and are nowadays widely applied in electric/electronic cast resins [44,45] and polyester fibers [46,47,48,49]. Some researchers suggested that the flame-retardant mechanism of DOPO and its derivatives can be explained by the release of low-molecular-weight phosphorous-containing species which are able to scavenge the H· and OH· radicals in the flame [50].

**Scheme 13:**
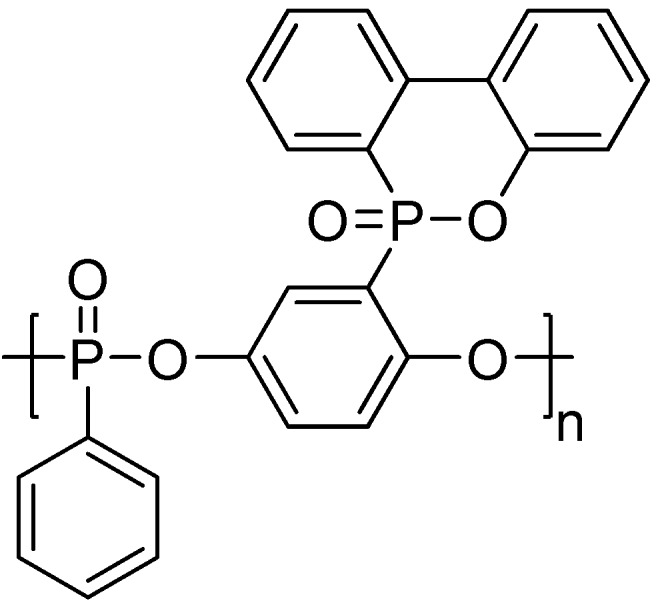
Chemical structure of poly(9-oxa-10-(2,5-dihyro-xyphenyl) phosphaphenanthrene-10-oxide) phenylphosphonate (PDPPP).

**Scheme 14:**
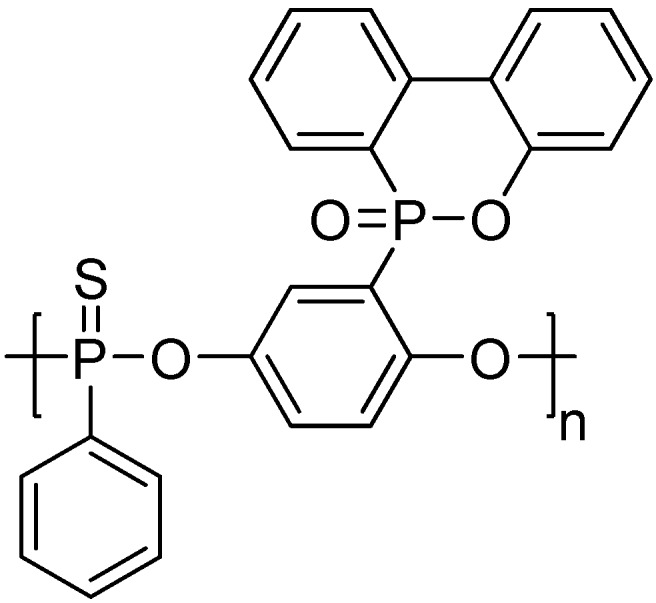
Chemical structure of poly(9-oxa-10-(2,5-dihyro-xyphenyl) phosphaphenanthrene-10-oxide) phenylthiophosphonate (PDPTP).

The high phosphorus content and rich aryl group structures of a novel main-chain/side-chain combined ArPPN (MS-ArPPN), named poly(9-oxa-10-(2,5-dihyro-xyphenyl) phosphaphenanthrene-10-oxide phenylphosphonate) (PDPPP, **Scheme 13**), synthesized by Wang and co-workers could contribute excellent flame retardancy to PET without a considerable decrease of mechanical properties [51]. The LOI value of the flame-retardant PET with 5 wt % PDPPP (P % as 0.70 wt % in the blend) reached 32.4 and the sample achieved V-0 during UL-94 testing. Epoxy resin and unsaturated polyester blended with PDPPP could also receive satisfactory flame retardancy. Furthermore, an aryl polyphosphonate similar to PDPPP, sulfur-containing aryl polyphosphonate, named poly(9-oxa-10-(2,5-dihyro-xyphenyl phosphaphenanthrene-10-oxide) phenylthiophosphonate) (PDPTP, **Scheme 14**), was synthesized by Wang *et al*. [52]. The flame-retardant action on PET was also investigated through FTIR, Py-GC/MS and Cone calorimetry. Results showed that the contents of flammable volatiles generated during combustion and pyrolysis were sharply reduced after PDPTP was added into PET matrix, which proved that the extensive pyrolysis of PET was retarded by the existence of PDPTP; however, PDPTP did not change the mechanism of pyrolysis of PET. According to the Cone results, the time to ignition was prolonged from 47 to 63 s after adding 10 wt % PDPTP into PET, which implied rather lower flammability. The PHRR value of PET was sharply decreased by 57% after adding PDPTP, conferring excellent flame retardancy on PET. Also, PDPTP was inclined to form char during combustion because the amount of char residue increases from 2.4 wt % for PET to 7.1 wt % for FR-PET, proving the excellent flame retardancy of PDPTP. Besides, PDPTP had good smoke suppression shown by the decrease of the specific extinction area after adding PDPTP. Therefore, PDPTP can manifest the flame retardation on PET both in the condensed phases and in gas phase. Furthermore, Ban *et al*. [53] investigated the dripping behavior of the flame-retardant PET containing PDPPP and PDPTP, respectively. For comparison, the authors employed the main-chain type PSTPP to prepare the flame-retardant PET. Detailed results are listed in Table 5. The authors suggested the introduction of sulfur element decreased the thermal stability of the MS-ArPPNs; and the weak P-C bond linking DOPO pendent group decreased it further. The decreased thermal stability could be helpful to aromatization and charring of the flame retardant before the sample was ignited; hence the three kinds of ArPPNs exhibited different dripping behaviors during the UL-94 test.

**Table 6:** Test results of flammability and melt-dripping behaviors of PET/ArPPN blends during the UL-94 test [53].

Sample	First ignition		Second ignition		UL-94 rating
	Burning time (s) ^a^	Observed dripping ^b^	Burning time (s)	Observed dripping	
PET/2% PDPTP	0	drip	2	drip	V-2
PET/5% PDPTP	0	heavy	0	heavy	V-0
PET/10% PDPTP	0	drip	0	drip	V-0
PET/2% PDPPP	0	drip	2	drip	V-2
PET/5% PDPPP	0	drip	0	drip	V-0
PET/8% PDPPP	0	drip	0	drip	V-0
PET/5% PDPPP/5% PSTPP	0	drip	0	drip	V-0
PET/2% PSTPP	>180	scarcely	none ^c^	none	NR
PET/5% PSTPP	>180	scarcely	none	none	NR
PET/10% PSTPP	>180	no	none	none	NR
PET/20% PSTPP	1	no	2	no	V-0

^a^ Average time to self-extinguishing after ignition; ^b^ Indicated that samples did (yes) or did not (no) drip onto the cotton patch underneath the bar during the UL-94 test. ^c^ There is no enough sample left after the first ignition, hence the second ignition cannot be carried out in that case.

## 4. Conclusions and Prospect

During two decades, polymer-type phosphonates (polyphosphonates, PPNs) have received increasing interest from both academia and industry. PPNs have excellent flame retardancy and transparency and suitable melting points or flow temperatures, which can match the processing temperatures of different polymers that are flame retarded. The melted flame retardants are very helpful to the compounding of flame retardant polymer systems. In addition, due to their polymeric nature, they will not migrate out of the matrices when used as additives. Moreover, in some polymer systems, the addition of PPNs can improve the polymers’ properties, such as heat distortion temperature. The development history of PPNs goes back in patent literature to 1948, but they were not commercialized because they had low strength, low glass transition temperature, and poor hydrolytic stability. In the 1980s, Bayer AG in Germany discovered a way to make PPNs with better properties, however, it was based on expensive ingredients that made it uneconomical [54]. Eventually Bayer dropped the development. In 2007, FRX Polymers, USA, announced that it commercialized a new family of aliphatic polyphosphonate homopolymers [55], copolymers [56,57] and branched polymers [58] as polymeric flame retardant additives for many plastics (*i.e.*, PET, PBT, poly(trimethylene terephthalate) (PTT), PC and arylonitrile-butadiene-styrene copolymer (ABS)) and as transparent, high flowing, non-burning specialty polymers [59].

In this article, a number of ArPPNs are summarized, and the potential mechanism for flame retardation is also discussed. Most of them exhibit condensed-phase activity on flame retardation; however, due to its bulky pendent DOPO group, MS-ArPPNs also show vapor-phase activity to a certain extent, which results in worse melt-dripping behaviors during combustion than PSTPP. Aside from these thermal stability constraints, good compatibility with the polyester melt and the absence of any detrimental effects on the spinnability are mandatory. Compared with the AlPPNs, ArPPNs received much more difficulty during commercialization; only a few Chinese companies such as Weili Flame-Retardant Chemicals, Chengdu, China [60], have announced that PSPPP has been commercialized until now. Although the bright future of ArPPN is foreseeable, the practical applications of ArPPNs are still underway.
